# Translation, validity and reliability of the Danish version of the Adolescent Insomnia Questionnaire

**DOI:** 10.12688/f1000research.25832.3

**Published:** 2022-04-01

**Authors:** Alessandro Andreucci, Christian Lund Straszek, Michael Skovdal Rathleff, Clara Guldhammer, Rocio de la Vega, Tonya M. Palermo

**Affiliations:** 1 Center for General Practice, Aalborg University, Aalborg, 9220, Denmark; 2 Department of Physiotherapy, University College of Northern Jutland (UCN), Aalborg, 9000, Denmark; 3 Department of Health Science and Technology, Faculty of Medicine, Aalborg University, Aalborg, 9000, Denmark; 4 Center for Child Health, Behavior and Development, Seattle Children's Research Institute, Seattle, WA, 98101, USA; 5 Department of Anesthesiology & Pain Medicine, University of Washington School of Medicine, Seattle, WA, 98101, USA

**Keywords:** Insomnia, Adolescent, Screening, Sleep, Translation, Validation

## Abstract

**Background**: The Adolescent Insomnia Questionnaire (AIQ), English version, is the only validated screening measure developed specifically to identify insomnia symptoms in adolescents. To date, no specific screening tool for adolescent insomnia is present in Danish language. The aim of this study was to translate and validate the AIQ in a sample of Danish adolescents.

**Methods**: The AIQ underwent a process of forward-backward translation and pilot testing. Subsequently, data were collected at baseline and two-week follow-up from adolescents aged 11-19, who completed both the AIQ and an available adult measure of insomnia (the Athens Insomnia Scale, AIS). The internal consistency, test-retest reliability and convergent validity were assessed. Exploratory factor analysis was conducted to identify the latent factors underlying the questionnaire.

**Results**: At baseline 185 adolescents (18% males and 82% females, mean age 16.0 years) and 102 (55.1%) at two-week follow-up completed the questionnaires. The AIQ showed excellent internal consistency for the total score (Cronbach's a: 0.88) and good convergent validity with the AIS total score (Pearson’s correlation value= 0.86, P<0.001). The test-retest reliability at two weeks was very satisfactory (ICC coefficient = 0.89; 95% CI 0.84, 0.92). Results from the exploratory factor analysis identified a three-model solution corresponding to the same three-model solution identified within the original development sample.

**Conclusions**: The Danish version of the AIQ demonstrated satisfactory psychometric properties in terms of internal consistency, test-retest reliability and validity, which supports its use as a screening tool for the identification of insomnia symptoms in adolescents, including Danish-speaking adolescents.

## Introduction

Insomnia is a common sleep disorder during adolescence that affects between 10 and 40% of adolescents, depending on the diagnostic criteria used ^1– 3^. Insomnia is associated with broad negative impact on emotional, social, cognitive and academic functioning ^2– 5^, and poor quality of life. There are also potential health problems associated with insomnia, such as anxiety, depression, obesity, substance abuse and chronic musculoskeletal pain ^2, 6– 9^. In addition, insomnia symptoms in adolescents may lead to use of sleep medications and other substances (e.g. alcohol, illicit drugs), which could result in additional risk for other disorders ^2^. Insomnia can persist into adulthood ^3^ with increased potential burden over the long-term ^10^. The estimated average 6-month cost (including direct and indirect costs) of insomnia in adults in the U.S.A. (2003 figures) was about $1,253 greater compared to individuals without insomnia ^11^. Insomnia is, therefore, an important public health issue ^2^.

The prevalence of insomnia during adolescence has increased during the last several decades ^7, 12^. It is hypothesized that this is due to increased availability of electronic devices in the bedroom and consumption of caffeinated beverages ^2, 7, 13, 14^. These changes in lifestyle affect the biological homeostasis, together with the hormonal changes occurring in a crucial developmental period such as puberty, impacting sleep patterns and resulting in changes in the sleep architecture ^15^. Additional changes related to the social domain, such as academic pressure and early start time for school, can contribute to insomnia symptoms ^2, 14, 15^. Altogether, these changes result in adolescents getting less sleep than needed. The identification of insomnia symptoms early on in life is extremely important ^7^. This would allow for early referral for intervention to address the symptoms and reduce potential health problems associated with insomnia. However, sleep disorders such as insomnia often go unrecognized in pediatric primary care ^2, 16^.

Pediatricians need a developmentally appropriate measure for the assessment of insomnia in adolescents that has adequate psychometric properties and high clinical utility (i.e., is brief, simple to use). In order to fill this gap, the Adolescent Insomnia Questionnaire (AIQ) has been recently developed to identify insomnia symptoms in adolescents and guide treatment decisions. This questionnaire was tested in a case-mix of 314 English-speaking American adolescents (11–18 years old) recruited from a sleep clinic, pain clinic, headache clinic and the community. In this heterogeneous sample of adolescents, the questionnaire showed good internal reliability, convergent and discriminant validity, and high criterion validity. However, it is not known if such a questionnaire will prove to be effective in other languages and cultural contexts such as Denmark.

To date, there is no valid insomnia questionnaire for adolescents available in Danish language. Due to the lack of validated tools for the assessment of adolescent insomnia, other measures originally developed for the assessment of insomnia in adult populations are used in Denmark, such as the Athens Insomnia Scale (AIS). However, the AIS has not undergone proper psychometric testing in a Danish adolescent population, and includes fewer items that do not capture the whole range of problems with sleep maintenance and sleep onset and also the specific impairments in the adolescents’ life (e.g. problems at school or with friends), which can be assessed with the AIQ. Testing the properties of the AIQ in an additional sample of adolescents in a different country, language and culture would provide additional support for the AIQ as an appropriate screening measure to assess insomnia in adolescents. Therefore, the aim of this study was to translate the Adolescent Insomnia Questionnaire into Danish and validate it in a sample of Danish adolescents attending a primary care clinic and from the community.

## Methods

### Study design

This is a prospective study with data collected at two time-points: baseline and two-week follow-up. Due to the non-interventional nature of the study and the regulation of the Scientific Ethics Committee for Region North Jutland, the study was exempt from ethical approval. Written informed consent was obtained at baseline and self-completed if participants were 15 years or older, otherwise it was completed by one parent/guardian if participants were younger than 15, and the minors provided their assent.

### Participant recruitment

Data were collected from adolescents aged 11–19 years old recruited from one general practice clinic in the city of Aalborg (n = 10, recruited in February 2020), Denmark and through social media advertisement (i.e. Facebook, n = 175, recruited in March/April 2020). Adolescents who attended primary care for any type of symptom/condition were provided with the questionnaire (together with the informed consent) to be completed in the waiting room of the general practice. Likewise, adolescents who responded to the Facebook advertisement were provided with the questionnaire (together with the informed consent) to be completed through a web application (RedCap) ^17^ accessible by a link in the advertisement. The Facebook post was tailored in order to be advertised to the population of interest (adolescents aged 11–19 and parents of adolescents) in the country of Denmark. After two weeks, an e-mail was sent to all the adolescents containing a link to a re-administration of the questionnaire to be completed at home in order to assess test-retest reliability. In both the baseline and follow-up questionnaire it was clearly stated that the questionnaire was intended to be completed by the adolescents. If participants did not complete the follow-up questionnaire, they were contacted by phone, SMS, or e-mail reminders. If four unsuccessful contact attempts were made or 10 days had passed without obtaining a response, no further contact was made.

### Measures

***Demographic information.*** The participants’ age and sex were collected.

***Adolescent Insomnia Questionnaire (AIQ).*** The AIQ ( Table 1) is a 13-item self-report screening measure of insomnia symptoms developed specifically for adolescents; it contains three subscales (sleep onset, sleep dissatisfaction and impairments, sleep maintenance) ^3^. The AIQ was validated in a sample of adolescents aged 11–18 years old with and without chronic pain conditions who were recruited from the community and clinical settings. The AIQ showed acceptable convergent (range .47–.88, p <.01) and discriminant (r = .06, p = .334) validity and strong reliability for both the total score (α = .91) and the subscale scores (α = .79 - .89) ^3^. A confirmatory factor analysis (CFA) revealed three factors consistent with the three subscales. Total scores range from 0 to 52, with higher scores indicating more severe insomnia symptoms. A provisional cut-off score of 15 for identifying insomnia was suggested following receiver-operator curve analysis of the development sample ^3^. The AIQ can be completed in approximately five minutes and it is relatively easy to score (there are only four reversed items) ^3^. The original English version of the AIQ was obtained through contact with the authors who developed the tool at the Seattle Children's Research Institute.

**Table 1.** Adolescent Insomnia Questionnaire, English version.

The following statements are about your sleep and possible difficulties with sleep. We would like to know more about your sleep during a **usual week**. If the last week was unusual for some reason, think about the most recent typical week. For each statement, mark the answer that best describes you.					
	Never	Almost Never	Sometimes	Often	Almost Always
1. I have difficulty falling asleep.	**0**	**1**	**2**	**3**	**4**
2. I wake up too early and cannot fall back asleep.	**0**	**1**	**2**	**3**	**4**
3. I am satisfied with my sleep.	**0**	**1**	**2**	**3**	**4**
4. I fall asleep quickly.	**0**	**1**	**2**	**3**	**4**
5. I feel sleepy or tired during the day.	**0**	**1**	**2**	**3**	**4**
6. It is hard for me to fall back to sleep when I wake up during the night.	**0**	**1**	**2**	**3**	**4**
7. It takes me more than a half hour to fall asleep.	**0**	**1**	**2**	**3**	**4**
8. I sleep through the night.	**0**	**1**	**2**	**3**	**4**
9. It is easy for me to settle down when it is time to go to sleep.	**0**	**1**	**2**	**3**	**4**
For the next statements, please think about how your sleep has affected you ***during the day*** in a **usual** **week**. If the last week was unusual for some reason, think about the most recent typical week. For each statement, mark the answer that best describes you.					
	**Never**	**Almost** **Never**	**Sometimes**	**Often**	**Almost** **Always**
1. I have trouble going to school because of sleep problems.	**0**	**1**	**2**	**3**	**4**
2. I have trouble paying attention in class or concentrating because of poor sleep.	**0**	**1**	**2**	**3**	**4**
3. I feel grumpy or sad because of poor sleep.	**0**	**1**	**2**	**3**	**4**
4. I have trouble doing things with friends because of poor sleep.	**0**	**1**	**2**	**3**	**4**

***Athens Insomnia Scale (AIS).*** The Athens Insomnia Scale (AIS) is an eight-item self-administered tool developed to assess insomnia severity in adults ^18^, which has also been used in adolescent populations in a few limited validation studies ^19, 20^. The AIS includes a variety of insomnia symptoms (difficulty with sleep initiation, awakenings during the night, early morning awakening, total sleep time, overall quality of sleep, sense of well-being during the day, physical and mental functioning during the day and sleepiness during the day) occurring on a frequency of at least three times per week during the last month ^18^. Each item can be rated with a number from 0 (no problem at all) to 3 (very serious problem). The resulting total score ranges from 0 (no sleep-related problems) to 24 (most severe degree of insomnia). The AIS showed good internal consistency (α = .89) and good test-retest reliability for both the total score (Pearson's correlation coefficient = .89) and for each item (Pearson's correlation coefficients = .70 - .86). The AIS also showed good external validity when compared to the Sleep Problems Scale (Pearson's correlation coefficient = .90) ^18^. The cut-off score for defining insomnia based on the AIS is 6 ^21^. A Danish translated version of the AIS has been previously used in a study conducted in a Danish general practice where participants >12 years old were assessed for insomnia symptoms ^22^.

### Stages of the project

The Adolescent Insomnia Questionnaire was translated to Danish by a panel composed by A.A., C.L.S., M.S.R., and M.A. Following translation, a preliminary pilot test was conducted and then the measure was validated in a larger sample of adolescents. Guidelines for the translation and validation of a questionnaire were followed ^23^.

***Translation of the questionnaire to Danish.*** A process of forward-backward translation of each item included in the AIQ from English to Danish was applied. This was done to ensure that the wording of the items in Danish was conceptually equivalent to the wording of the items in English. Two forward translations were initially produced by two native Danish researchers (C.L.S. and M.S.R.). The two translations were then discussed within the research team until a final decision on the translation of each item was reached. This version was then translated back to English (backward translation) and compared to the original version in English to assess potential differences between the two versions. The backward translation process was carried out by a bilingual researcher native in English. During the process of translation, items of the AIQ were properly worded for the age of adolescents in order to ensure comprehensibility and face validity.

***Preliminary pilot testing of the questionnaire.*** After the initial translation stage described above, the AIQ was tested with volunteer participants (n = 11, age range 10 - 19) recruited through advertisement of the study on Facebook. Children and adolescents who were interested in the study (or their parents) contacted the primary investigator of this study (A.A.), and a date was arranged for testing the tool and carrying out cognitive interviews with a research assistant (C.G). Study procedures were carried out at the Center for General Practice at Aalborg University, and participants were given a cinema ticket as a reward for participating in the cognitive interviews. The initial translated version of the AIQ was delivered to participants who were instructed to complete it by themselves. After completion of the questionnaire, cognitive interviews were carried out to assess the comprehensibility of the items. The aim was to improve the face and content validity of the tool at this stage. The feedback received through cognitive interviews indicated that no change to the Danish translated version of the AIQ was needed ( Table 2). The translated version was subsequently validated through the process described below.

**Table 2.** Adolescent Insomnia Questionnaire, Danish version.

De følgende udsagn handler om din søvn og mulige problemer i forbindelse med din søvn. Vi vil gerne vide mere om din søvn igennem en **normal uge**. Hvis sidste uge har været anderledes af en eller anden grund, så tænk tilbage på den seneste normale uge. Markér det svar, som passer bedst.					
	Aldrig	Næsten aldrig	Nogle gange	Ofte	Næsten altid
1. Jeg har svært ved at falde i søvn.	**0**	**1**	**2**	**3**	**4**
2. Jeg vågner for tidligt og kan ikke falde i søvn igen.	**0**	**1**	**2**	**3**	**4**
3. Jeg er tilfreds med min søvn.	**0**	**1**	**2**	**3**	**4**
4. Jeg falder hurtigt i søvn.	**0**	**1**	**2**	**3**	**4**
5. Jeg føler mig søvnig eller træt i løbet af dagen.	**0**	**1**	**2**	**3**	**4**
6. Det er svært for mig at falde i søvn igen, når jeg vågner op om natten.	**0**	**1**	**2**	**3**	**4**
7. Det tager mig mere end en halv time at falde i søvn.	**0**	**1**	**2**	**3**	**4**
8. Jeg sover igennem hele natten.	**0**	**1**	**2**	**3**	**4**
9. Det er nemt for mig at falde til ro, når det er tid til sove.	**0**	**1**	**2**	**3**	**4**
Ved de næste udsagn skal du tænke på hvordan din søvn påvirker dig *i løbet af dagen* i en **normal uge**. Hvis sidste uge har været anderledes af en eller anden grund, så tænk tilbage på den seneste normale uge. Markér det svar, som passer bedst.					
	**Aldrig**	**Næsten** ** aldrig**	**Nogle** **gange**	**Ofte**	**Næsten** **altid**
10. Jeg har svært ved at gå i skole, fordi jeg sover dårligt.	**0**	**1**	**2**	**3**	**4**
11. Jeg har svært ved at koncentrere mig eller høre efter i skolen, fordi jeg sover dårligt.	**0**	**1**	**2**	**3**	**4**
12. Jeg føler mig gnaven eller ked af det på grund af dårlig søvn.	**0**	**1**	**2**	**3**	**4**
13. Jeg har svært ved at lave ting sammen med mine venner, fordi jeg sover dårligt.	**0**	**1**	**2**	**3**	**4**

***Validation of the questionnaire.*** Several parameters were assessed during the validation of the questionnaire. Cronbach's α coefficient was calculated to assess the internal consistency of the AIQ total score and of the AIQ subscales. Test-retest reliability was assessed by comparing the responses to each item of the AIQ between baseline and two-week follow-up in order to evaluate the short-term stability of the questionnaire in adolescents ^24, 25^. Convergent validity was assessed by comparing the scores obtained with the AIQ with those obtained with the AIS. Exploratory factor analysis was conducted in order to identify the latent factors underlying the questionnaire, and compare them to the original English version of the measure ^26^.

### Statistical analysis

Descriptive analysis of the study sample was performed. Results are shown as means and standard deviations (SD) or as counts (%) depending on the type of variable (continuous or categorical). T-tests or Pearson *χ* ^2^-tests were used to compare groups on continuous and categorical variables, respectively. Cronbach's α for the AIQ total score and for the AIQ subscales (sleep onset subscale, sleep maintenance subscale, sleep dissatisfaction and impairments subscale) was calculated in order to assess internal consistency reliability. Test-retest stability was evaluated by assessing the relationship between the AIQ total score and subscale scores between baseline and follow-up by means of the intraclass correlation (ICC) coefficient, using a two-way mixed-effects model. If the ICC values are <0.5, this is indicative of poor reliability, while values between 0.5 and 0.75 show moderate reliability. ICC values between 0.75 and 0.9 show good reliability, and values > 0.90 excellent reliability ^25^. Limits of agreements (LoA) were also used to express the agreement between the two measurements (i.e. baseline vs. follow-up). The LoA represent the mean difference between the two measurements ±1.96 times the standard deviation of the differences ^27^. Convergent validity was assessed by calculating Pearson correlations between the AIS total score and the AIQ total score (and scores for subscales). Exploratory factor analysis (EFA) using principal factor extraction and oblique rotation was conducted to identify the factor loadings. Results of EFA were compared to those from the original development sample. The Kaiser-Meyer-Olkin (KMO) Measure of Sampling Adequacy test and Bartlett’s Test of Sphericity were performed to assess the suitability of the data for factor analysis. Analysis were conducted with STATA version 15.0.

## Results

### Participant characteristics

Participant characteristics are shown in Table 3. The sample included 185 participants at baseline, 33 (18%) males and 151 (82%) females. One participant did not report their sex. The mean age of participants was 16.0 years (± 1.4, range 11–19 years). One-hundred and two participants (55.1%) completed the questionnaires at two-week follow-up. The average AIQ score at baseline was 30.14 (± 8.90). Females had a statistically significant (31.1 ± 8.1, p = 0.003) higher mean score than males (26.0 ± 10.8). Higher AIQ scores were significantly associated with older age (linear regression coefficient = 1.48; 95% CI 0.58, 2.39; P = 0.001). Ninety-two percent of the sample (N = 169) had AIQ values above the suggested cut-off of 15 for defining insomnia symptoms. The average AIS total score was 11.4 (± 4.8), and 83% of the sample (N = 169) had AIS values higher than the cutoff of 6 for defining insomnia symptoms. Thus, this sample represented those adolescents with high levels of insomnia symptoms.

**Table 3.** Descriptive characteristics at baseline and two-week follow-up.

*Baseline*			
	Males (n=33)	Females (n=151)	Total (n=185)
Age (years), mean ± SD	16.0 (± 1.9)	15.9 (± 1.3)	16.0 (± 1.4)
AIQ score, mean ± SD	26.0 (± 10.8)	31.1 (± 8.1)	30.1 (± 8.9)
AIS score, mean ± SD	9.4 (± 5.9)	11.9 (± 4.4)	11.4 (± 4.8)
*Two-week follow-up*			
	Males (n=13)	Females (n=89)	Total (n=102)
Age (years), mean ± SD	15.7 (± 2.4)	15.8 (± 1.4)	15.8 (± 1.6)
AIQ score, mean ± SD	23.9 (± 12.1)	30.6 (± 8.2)	29.7 (± 9.0)
AIS score, mean ± SD	9.7 (± 6.4)	12.0 (± 5.1)	11.7 (± 5.3)

SD = standard deviation

### Internal consistency of the AIQ

The internal consistency of the AIQ was calculated for both the AIQ total score and the AIQ subscales ( Table 4). The internal consistency for the AIQ total score was excellent (Cronbach's α: 0.88). The internal consistency for both the AIQ sleep onset subscale (items 1, 4, 7, 9) and AIQ sleep dissatisfaction and impairments subscale (items 3, 5, 10, 11, 12, 13) was excellent as well (Cronbach's α: 0.84 and 0.87, respectively). The internal consistency for the AIQ sleep maintenance subscale (items 2, 6, 8) was slightly lower (Cronbach's α: 0.73).

**Table 4.** Internal consistency and validity.

Internal consistency	Cronbach's α:
AIQ total score	0.88 *
Sleep onset subscale	0.84 *
Sleep impairment subscale	0.87 *
Sleep maintenance subscale	0.73 *
Validity (assessed against the AIS total score)	Pearson’s correlation value
AIQ total score	0.86 *
Sleep onset subscale	0.59 *
Sleep impairment subscale	0.83 *
Sleep maintenance subscale	0.58 *

AIQ = Adolescent Insomnia Questionnaire AIS = Athens Insomnia Scale * = statistically significant

### Validity of the AIQ

The convergent validity of the AIQ was evaluated using correlations with the total score of the AIS ( Table 4). A large, positive significant correlation between the AIQ total score and the AIS total score was found (Pearson’s correlation value= 0.86, P<0.001). The positive correlation between the AIQ total score and the AIS is also illustrated in Supplementary Figure 1. Subscale scores of the AIS were also significantly related to the AIS with the largest correlation between the sleep dissatisfaction and impairment subscale (Pearson’s correlation value = 0.83, P<0.001) and the AIS and smaller correlations for the sleep onset subscale (Pearson’s correlation value = 0.59, P<0.001) and the sleep maintenance subscale (Pearson’s correlation value = 0.58, P<0.001).

### Test-retest reliability of the AIQ

Results of the test-retest reliability analysis at two-weeks are shown in Table 5. The ICC coefficient for the AIQ total score was 0.89 (95% CI 0.84, 0.92), while it was 0.86 (95% CI 0.79, 0.90) for the sleep onset subscale, 0.86 (95% CI 0.80, 0.90) for the sleep impairment subscale and 0.80 (95% CI 0.71, 0.86) for the sleep maintenance subscale. These values demonstrate strong test-retest reliability.

**Table 5.** Results of the test-retest reliability analysis.

	ICC	95% CI
AIQ total score	0.89	0.84, 0.92
Sleep onset subscale	0.86	0.79, 0.90
Sleep impairment subscale	0.86	0.80, 0.90
Sleep maintenance subscale	0.80	0.71, 0.86

ICC = intraclass correlation coefficient 95% CI = 95% confidence intervals

### Limits of agreement

The limits of agreement calculation showed limits of agreement from – 7.371 to 9.553 for the AIQ total score, and a mean difference of 1.091 (95% CI: 0.247, 1.935). This shows a good agreement between the two measurements (i.e. baseline vs. follow-up), as there was a mean difference of 1 point in the AIQ score, which can be considered small on a scale ranging from 0 to 52.

### Exploratory factor analysis

Results from EFA showed a three-factor solution that supported the model identified in the original development sample and accounted for 65.45% of the variance among items. Values of the rotated individual items ranged from 0.60 to 0.84 ( Table 6) and were similar to those identified within the original development sample. Factor cross-loadings of the AIQ items are provided in Supplementary Table 1. The value for the KMO test was 0.877 and the p-value for the Bartlett test of sphericity was <0.001, indicating that the sample is adequate for the Exploratory Factor Analysis.

**Table 6.** Factor loadings in the three-factor model (13 items).

*Factor 1: Sleep dissatisfaction and impairments*	Rotated factor loadings
Item 11: I have trouble paying attention in class or concentrating because of poor sleep	0.83
Item 10: I have trouble going to school because of sleep problems	0.82
Item 12: I feel grumpy or sad because of poor sleep.	0.63
Item 13: I have trouble doing things with friends because of poor sleep. Item 5: I feel sleepy or tired during the day. Item 3: I am satisfied with my sleep. *	0.60 0.76 0.61
*Factor 2: Sleep onset*	
Item 1: I have difficulty falling asleep.	0.78
Item 4: I fall asleep quickly. *	0.84
Item 7: It takes me more than a half hour to fall asleep.	0.80
Item 9: It is easy for me to settle down when it is time to go to sleep. *	0.73
*Factor 3: Sleep maintenance*	
Item 2: I wake up too early and cannot fall back asleep.	0.83
Item 6: It is hard for me to fall back to sleep when I wake up during the night	0.73
Item 8: I sleep through the night. *	0.79

* = Item is reverse scored.

## Discussion

The objective of this study was to translate and validate a self-report screening measure of insomnia symptoms for adolescents, the AIQ, to Danish, providing additional support for the AIQ and allowing its use in a broader population. The translated AIQ showed good content validity, good to excellent internal consistency, and very satisfactory test-retest reliability. The translated questionnaire demonstrated good convergent validity as evidenced by positive strong correlations between the AIS total score and the AIQ total score. Exploratory factor analysis resulted in a three-model solution corresponding to the same three-model solution identified within the original development sample ^3^, providing additional support for construct validity. Altogether the findings demonstrated that the AIQ has excellent reliability and validity and high clinical utility in a Danish translation of the measure.

According to the Cohen criteria for establishing the quality of pediatric questionnaires ^28^ the AIQ would be considered a “well-established” measure; this means it has been assessed by at least two studies by two different research teams, which have both evaluated the psychometric properties with information on validity and reliability and have provided details that allow critical evaluation and replication. Therefore, the AIQ has the potential to be used for the assessment of insomnia symptoms in both research and clinical settings. This might be relevant especially for the detection of adolescents’ insomnia symptoms in the primary care setting, where they are usually under-diagnosed ^1, 16^. However, further studies of the AIQ may help to provide evidence of validity in other settings with different populations. The original version of the AIQ (in English) was tested in a mixed population of adolescents from a pediatric sleep clinic, a pediatric pain clinic, a pediatric headache clinic and healthy adolescents recruited from the community. Further data across the full range of demographics (e.g. a sample with equal proportions of males and females, younger adolescents) and patients with conditions that are associated with insomnia (e.g. chronic pain, depression, other sleep disorders) ^29^ are needed before a cut-off for the Danish version of the AIQ can be established. In addition, other studies where the score obtained with the AIQ is compared to other sleep tools such as sleep diaries, which might also be filled electronically ^30^, might provide more insights on the stability of the AIQ on a longer time-period.

The results of the study should be interpreted in light of several limitations. First, there are limitations in terms of external validity ^31^. The majority of participants in this sample were female (82%), most of them (N = 175) were recruited through a Facebook advertisement, and the sample had severe insomnia symptoms, as shown by the high AIQ and AIS total score values, which were both well above the respective proposed cut-offs for clinically significant insomnia symptoms. This might be due to a self-selection bias of participants responding to an ad related to sleep research in social media. Indeed, in our sample most adolescents were recruited through a Facebook post, which might have attracted adolescents with severe symptoms of insomnia. In addition, a recent meta-analysis showed a high prevalence of sleep disturbances, especially in children and adolescents (46% prevalence) during the COVID-19 pandemic ^32^, which is the period when online recruitment occurred. This might have increased the prevalence of insomnia symptoms in the sample. While this highlights the relevance and need for further identifying and treating insomnia in adolescents, it does limit the psychometric evaluation. Therefore, further research with other pediatric samples with a broader range of insomnia symptoms (e.g. general population, medical or psychiatric samples) is needed to assess the stability of the findings. Second, a CFA was not performed as our aim was not to confirm that the AIQ factor structure is the same in a different language, but to explore what the factor structure would be in such language, without making assumptions, given that the languages and culture are different. In addition, the AIQ is still in an early phase of instrument development. Therefore, future plans will be to conduct a CFA once there is further validity evidence on the internal structure of the scale from similar populations and contexts. Third, in order to reduce participant burden very limited demographic information were collected. Because race and ethnicity were not collected it was not possible to assess whether the Danish translation of the AIQ performs well in ethnic minority populations. Further validation of the AIQ is needed, and may benefit from using item response theory to establish a link between the properties of items on the AIQ, subgroups of individuals responding to these items, and the underlying trait being measured. We also do not have information on adolescent’s concurrent health or mental health conditions and therefore are limited in understanding whether responses differ based on underlying health conditions. Future studies should include populations with specific conditions (e.g. chronic pain, depression) or other sleep disorders that might be comorbid with insomnia ^33, 34^, as these patients might be at higher risk of concurrent severe insomnia symptoms ^6^. Finally, adolescence is a period where changes in bedtime occur due to both biological and social factors ^7^, which, together with the effect of the inborn chronotype, might result in a social jetlag and consequential reduced sleep duration ^35^. Therefore, future studies should also try to measure the social jetlag that the adolescent might be experiencing.

In summary, the AIQ is a tool that can potentially fill the need for a validated, brief, screening measure for insomnia symptoms in adolescents ^30, 36^. It is available now translated in Danish and has shown good psychometric properties that allow for the use in Danish research and clinical settings. The AIQ can be used for the identification of adolescents with insomnia symptoms who can subsequently be referred for sleep intervention such as cognitive-behavioural therapy for insomnia for improvement of the condition early on ^6, 37^. In addition, the findings confirm the three‐factor structure identified in the development sample.

## Conclusions

The AIQ was translated to Danish and demonstrated satisfactory psychometric properties in terms of internal consistency, test-retest reliability and validity, which supports its use as a screening tool for the identification of insomnia symptoms in adolescents. Future studies for the exploration of its validity in populations with specific conditions and potentially different levels of insomnia symptoms are needed.

## Abbreviations

AIQ = Adolescent Insomnia Questionnaire

AIS = Athens Insomnia Scale

## Data availability statement

Data underlying the study are available at https://doi.org/10.7910/DVN/VQFT9W.

### Extended data

Supplementary Figure 1 and Supplementary Table 1 are available as extended data at https://doi.org/10.7910/DVN/VQFT9W.
