# Supplementary material for: Translation, validity and reliability of the Danish version of the Adolescent Insomnia Questionnaire
Source: F1000Res. 2022 May 3;9:1029. Originally published 2020 Aug 24. [Version 4] doi: 10.12688/f1000research.25832.4 (PMC9069172; doi:10.12688/f1000research.25832.4)
Supplement: Peer review history

# Peer review: Reviewer response for version 4

**DOI:** 10.5256/f1000research.126768.r136584

**Published:** 2022-05-04

**Authors:** Jefferson Santos

**Affiliations:** 1 Human Chronobiology Laboratory, Department of Physiology, Biological Sciences Sector, Federal University of Paraná, Curitiba, Brazil

The issue about "inborn" chronotypes has been satisfied in the new version of the article.

Is the work clearly and accurately presented and does it cite the current literature?

Yes

If applicable, is the statistical analysis and its interpretation appropriate?

Yes

Are all the source data underlying the results available to ensure full reproducibility?

Yes

Is the study design appropriate and is the work technically sound?

Yes

Are the conclusions drawn adequately supported by the results?

Yes

Are sufficient details of methods and analysis provided to allow replication by others?

Yes

Reviewer Expertise:

Chronobiology, Sleep Research

I confirm that I have read this submission and believe that I have an appropriate level of expertise to confirm that it is of an acceptable scientific standard.

# Peer review: Reviewer response for version 3

**DOI:** 10.5256/f1000research.122999.r129629

**Published:** 2022-04-07

**Authors:** Martin Ulander

**Affiliations:** 1 Department of Clinical Neurophysiology, Linköping University Hospital, Linköping, Sweden

Dear authors,

 I have read the updated version of the manuscript and I am satisfied with the responses to my comments and the changes that have been made to the article. The authors may want to add to the Methods section, Statistical analysis subsection, that they used the Kaiser criterion (eigenvalue > 1) to identify the number of factors to extract in the factor analysis. I have nothing to object to this, but as there are several methods described in the factor analysis literature to choose the number of factors to extract, and as they do not always reach the same conclusion, it is advisable to state which method was used. Other than that admittedly minor point, I see no need for further changes.

Is the work clearly and accurately presented and does it cite the current literature?

Yes

If applicable, is the statistical analysis and its interpretation appropriate?

Partly

Are all the source data underlying the results available to ensure full reproducibility?

Partly

Is the study design appropriate and is the work technically sound?

Yes

Are the conclusions drawn adequately supported by the results?

Yes

Are sufficient details of methods and analysis provided to allow replication by others?

Yes

Reviewer Expertise:

Sleep medicine, especially with regard to questionnaire development, translation and validation

I confirm that I have read this submission and believe that I have an appropriate level of expertise to confirm that it is of an acceptable scientific standard.

# Peer review: Reviewer response for version 2

**DOI:** 10.5256/f1000research.59059.r122851

**Published:** 2022-03-03

**Authors:** Martin Ulander

**Affiliations:** 1 Department of Clinical Neurophysiology, Linköping University Hospital, Linköping, Sweden

The study describes the translation and initial validation of the Adolescent Insomnia Questionnaire (AIQ) from English to Danish. There is a general lack of well-validated insomnia questionnaires for children and adults into Nordic languages, and from that perspective, the study represents a welcome addition. The main strength of the study is the clear and sound translation process. The main weakness is that most of the respondents were recruited via social media, which increases the risk of self-selection bias and means that less clinically relevant background information about the respondents is available. It should also be noted that the study only represents the initial steps needed for a full psychometric evaluation of the translated version.

**Introduction**

 The introduction is well-written and covers the most important areas.

**Methods, participant recruitment subsection: **What considerations were made concerning the number of subjects in the study?

**Methods, Stages of the project subsection:** The process seems adequate and is well described in the manuscript. As far as I can tell, given that I am not a native Danish speaker, but given that written Swedish (which is my native language) and written Danish are fairly similar, it seems like the translated version accurately conveys the meaning of the original English version, in that there is nothing obviously questionable about the translations.

**Methods, statistics subsection: **What criteria did you use to decide for a three-factor solution in the EFA rather than any other number of factors?

**Discussion**

 While selection bias due to the participant recruitment process is clearly a reasonable cause of the high prevalence of insomnia symptoms in the paper, I notice that data collection took place in the winter/spring of 2020, that is, in the beginning of the pandemic. Could this have affected the subjects and the prevalence of insomnia symptoms? I think this point could be raised in the discussion.

 Regarding future evaluation and validation of the Danish version of the questionnaire, all suggestions by the authors are advisable. I would like to add, as a further aim for future research, to see how the Danish AIQ performs in patients with other sleep disorders than insomnia, ie not only chronic pain and depression. Even as it is meant for insomnia patients, sleep disorders are often comorbid and it is reasonable to assume that some of the items of the AIQ will be affected by the presence of comorbid sleep disorders. Several of the items are worded in such a way that adolescents with other sleep disorders than insomnia are likely to score high on them as well.

 You raise the point of examining how the AIQ performs in various subgroups defined by e.g., ethnicity. I would suggest considering to also use item response theoretic methods to assess the AIQ including for example differential item functioning.

**Original data**

 All original data are available, but it is not always clear how to interpret the data. For example, age seems to range between 0 and 8. The item variables are calles ”aiq_item_n”, ”aiq_item_n_fo” (which I assume is follow-up), ”new_aiq_item_n” and ”new_aiq_item_n_fo”. What do the ”new” AIQ item scores represent?

 In summary, my suggestions to the authors are:

- Please clarify what, if any, considerations were made when deciding the number of participants.
- Please clarify on what criteria you based the decision to extract three factors in the EFA.
- Please add the need to assess how AIQ performs in patients with other sleep disorders as a point for future research. Regarding the possible effect of the pandemic, please add it if you consider it to be a possible contributing factor to the high prevalence of insomnia symptoms.
- If it is possible to add an extra supplementary file describing what the different variables in the data file represent and how they are coded, please do so as well.

Is the work clearly and accurately presented and does it cite the current literature?

Yes

If applicable, is the statistical analysis and its interpretation appropriate?

Partly

Are all the source data underlying the results available to ensure full reproducibility?

Partly

Is the study design appropriate and is the work technically sound?

Yes

Are the conclusions drawn adequately supported by the results?

Yes

Are sufficient details of methods and analysis provided to allow replication by others?

Yes

Reviewer Expertise:

Sleep medicine, especially with regard to questionnaire development, translation and validation

I confirm that I have read this submission and believe that I have an appropriate level of expertise to confirm that it is of an acceptable scientific standard, however I have significant reservations, as outlined above.

# Response

**Published:** 2022-03-29

**Authors:** Alessandro Andreucci

**Affiliations:** Center for General Practice at Aalborg University, Denmark

The study describes the translation and initial validation of the Adolescent Insomnia Questionnaire (AIQ) from English to Danish. There is a general lack of well-validated insomnia questionnaires for children and adults into Nordic languages, and from that perspective, the study represents a welcome addition. The main strength of the study is the clear and sound translation process. The main weakness is that most of the respondents were recruited via social media, which increases the risk of self-selection bias and means that less clinically relevant background information about the respondents is available. It should also be noted that the study only represents the initial steps needed for a full psychometric evaluation of the translated version.

*Response: We thank the reviewer for the valuable feedback provided. We have* *addressed the comments and changed the manuscript, which we feel is now improved. Individual responses for each specific comment are outlined below*.

**Introduction**

 The introduction is well-written and covers the most important areas.

*Response: We thank the reviewer for the comment.*

**Methods, participant recruitment subsection: **What considerations were made concerning the number of subjects in the study?

*Response: Unfortunately, we were limited by the special conditions that occurred (i.e., onset of COVID-19 pandemic) at the time of recruitment of participants to the study. The original English version of the Adolescent Insomnia Questionnaire was developed using a bigger sample of adolescents (n=* *315) and we acknowledge that a bigger sample size would have been beneficial for the analysis and external validity. However, both the Kaiser-Meyer-Olkin (KMO) Measure of Sampling Adequacy test and Bartlett’s Test of Sphericity showed that the sample was adequate for the Exploratory Factor Analysis.*

**Methods, Stages of the project subsection:** The process seems adequate and is well described in the manuscript. As far as I can tell, given that I am not a native Danish speaker, but given that written Swedish (which is my native language) and written Danish are fairly similar, it seems like the translated version accurately conveys the meaning of the original English version, in that there is nothing obviously questionable about the translations.

*Response: We thank the reviewer for the comment.*

**Methods, statistics subsection: **What criteria did you use to decide for a three-factor solution in the EFA rather than any other number of factors?

*Response: By performing the exploratory factor analysis we obtained estimates of eigenvalues and the cumulative proportion of explained variance. According to the Kaiser criterion (Osborne et al., 2011), only factors that have eigenvalues >1 should be selected for obtaining the optimal number of factors in the factor analysis. In our analysis, there were only three factors with eigenvalues >1 as shown in the table linked here.*

**Discussion**

 While selection bias due to the participant recruitment process is clearly a reasonable cause of the high prevalence of insomnia symptoms in the paper, I notice that data collection took place in the winter/spring of 2020, that is, in the beginning of the pandemic. Could this have affected the subjects and the prevalence of insomnia symptoms? I think this point could be raised in the discussion.

*Response: We thank the reviewer for the very insightful comment and have now modified the discussion section as follows:*

*“This might be due to a self-selection bias of participants responding to an ad related to sleep research in social media. Indeed, in our sample most adolescents were recruited through a Facebook post, which might have attracted adolescents with severe symptoms of insomnia. In addition, a recent meta-analysis showed a high prevalence of sleep disturbances, especially in children and adolescents (46% prevalence) during the COVID-19 pandemic *(Jahrami et al., 2022) *, which is the period when online recruitment occurred. This might have increased the prevalence of insomnia symptoms in the sample”*

 Regarding future evaluation and validation of the Danish version of the questionnaire, all suggestions by the authors are advisable. I would like to add, as a further aim for future research, to see how the Danish AIQ performs in patients with other sleep disorders than insomnia, ie not only chronic pain and depression. Even as it is meant for insomnia patients, sleep disorders are often comorbid and it is reasonable to assume that some of the items of the AIQ will be affected by the presence of comorbid sleep disorders. Several of the items are worded in such a way that adolescents with other sleep disorders than insomnia are likely to score high on them as well.

*Response: We thank the reviewer for the very insightful comment and have now modified the discussion section as follows:*

*”Future studies should include populations with specific conditions (e.g. chronic pain, depression)  or other sleep disorders that might be comorbid with insomnia (Meltzer et al., 2014; Nunes and Bruni, 2015*) *, as these patients might be at higher risk of concurrent severe insomnia symptoms”*

 You raise the point of examining how the AIQ performs in various subgroups defined by e.g., ethnicity. I would suggest considering to also use item response theoretic methods to assess the AIQ including for example differential item functioning.

*Response: We thank the reviewer for the very insightful comment and have now modified the discussion section as follows:*

*”Further validation of the AIQ is needed, and may benefit from using item response theory to establish a link between the properties of items on the AIQ, subgroups of individuals responding to these items, and the underlying trait being measured”*

**Original data**

 All original data are available, but it is not always clear how to interpret the data. For example, age seems to range between 0 and 8. The item variables are calles ”aiq_item_n”, ”aiq_item_n_fo” (which I assume is follow-up), ”new_aiq_item_n” and ”new_aiq_item_n_fo”. What do the ”new” AIQ item scores represent?

*Response: We have now deposited a clean updated dataset containing only the variables used for the analysis and a supplementary file in the repository where the dataset is located with a description of what each variable in the dataset represents.*

**References**

 Jahrami, H.A., Alhaj, O.A., Humood, A.M., Alenezi, A.F., Fekih-Romdhane, F., AlRasheed, M.M., Saif, Z.Q., Bragazzi, N.L., Pandi-Perumal, S.R., BaHammam, A.S., Vitiello, M. V. (2022). Sleep disturbances during the COVID-19 pandemic: A systematic review, meta-analysis, and meta-regression. *Sleep Med Rev* 62, 101591.

 Meltzer, L.J., Plaufcan, M.R., Thomas, J.H., Mindell, J.A. (2014). Sleep problems and sleep disorders in pediatric primary care: treatment recommendations, persistence, and health care utilization. *J Clin Sleep Med* 10, 421–426.

 Nunes, M.L., Bruni, O. (2015). IInsomnia in childhood and adolescence: clinical aspects, diagnosis, and therapeutic approach. *J Pediatr (Rio J)* 91, S26–S35.

 Osborne, J.W., Costello, A.B., Kellow, J.T. (2011). *Best Practices in Exploratory Factor Analysis*.

# Peer review: Reviewer response for version 2

**DOI:** 10.5256/f1000research.59059.r91109

**Published:** 2021-09-07

**Authors:** Jefferson Santos

**Affiliations:** 1 Human Chronobiology Laboratory, Department of Physiology, Biological Sciences Sector, Federal University of Paraná, Curitiba, Brazil

Dear authors,

 Corrections were done very well including the pivotal information about KMO and Bartlett's test measures. I have only one issue to highlight in the limitation text.

 The term "inborn chronotype" is very controversial because there is so much evidence about the role of environmental factors in circadian preference construction. For example, there are bimodal individuals who have a flexible circadian preference adopting morning or evening preferences according to your need. Therefore, I would suggest changing this part of the text to avoid any future problems with this concept.

Is the work clearly and accurately presented and does it cite the current literature?

Yes

If applicable, is the statistical analysis and its interpretation appropriate?

Yes

Are all the source data underlying the results available to ensure full reproducibility?

Yes

Is the study design appropriate and is the work technically sound?

Yes

Are the conclusions drawn adequately supported by the results?

Yes

Are sufficient details of methods and analysis provided to allow replication by others?

Yes

Reviewer Expertise:

Chronobiology, Adolescent Sleep

I confirm that I have read this submission and believe that I have an appropriate level of expertise to confirm that it is of an acceptable scientific standard, however I have significant reservations, as outlined above.

# Response

**Published:** 2022-04-08

**Authors:** Alessandro Andreucci

**Affiliations:** Center for General Practice at Aalborg University, Denmark

Dear Reviewer,

 Thank you very much for your insightful comment.

 We have now modified the text in the discussion by deleting the part regarding inborn chronotypes. The text now reads:

 "Finally, adolescence is a period where changes in bedtime occur due to both biological and social factors, which might result in a social jetlag and consequential reduced sleep duration."

 We hope this version is now acceptable for publication.

# Peer review: Reviewer response for version 1

**DOI:** 10.5256/f1000research.28509.r88082

**Published:** 2021-07-06

**Authors:** Jefferson Santos

**Affiliations:** 1 Human Chronobiology Laboratory, Department of Physiology, Biological Sciences Sector, Federal University of Paraná, Curitiba, Brazil

The research article achieved the aim to provide a good insomnia measurement to the adolescent population. The text is objective and the procedures are adequate to questionnaire validity and reliability. 

 Therefore, I would like to point two questions in order to improve the quality of the manuscript. 

**Major point:**

- Only exploratory factor analysis was performed. The factor loadings are important to comprehend the contribution of each item into the three-factor model extracted by EFA, however, the CFA is also important to measure the factor latent structure model of the AIQ. I suggest that CFA should be included in the analysis as well as the graphic demonstration of the CFA. Lastly, it would be recommended to report the KMO and Bartlett tests to show the suitability of the sample for factor analysis in EFA and the model adjustment indexes in CFA (RMSEA, CFI, GFI, and TLI). These values ensure robustness to the models.

**Minor points:**

- In table 6, the rotated factor loading of item 1 is missing. I suggest including the lack of a social jetlag measurement as a limitation of the study. It would be relevant information to better sample description.

Is the work clearly and accurately presented and does it cite the current literature?

Yes

If applicable, is the statistical analysis and its interpretation appropriate?

Yes

Are all the source data underlying the results available to ensure full reproducibility?

Yes

Is the study design appropriate and is the work technically sound?

Yes

Are the conclusions drawn adequately supported by the results?

Yes

Are sufficient details of methods and analysis provided to allow replication by others?

Yes

Reviewer Expertise:

Chronobiology, Adolescent Sleep

I confirm that I have read this submission and believe that I have an appropriate level of expertise to confirm that it is of an acceptable scientific standard, however I have significant reservations, as outlined above.

# Response

**Published:** 2021-07-27

**Authors:** Alessandro Andreucci

**Affiliations:** Center for General Practice at Aalborg University, Denmark

Dear Editorial Team, F1000 Research,

 We were delighted that the Reviewer of our submission " Translation, validity and reliability of the Danish version of the Adolescent Insomnia Questionnaire " (ID: 25832) provided us with their very helpful feedback on our paper. This has given us the opportunity to apply changes that has led to an improvement of the paper.

 Following the recommendations of the reviewer we have now modified the manuscript. We have set out below a response to the Reviewer’s specific comments, outlining point by point how we addressed the comments and changed the manuscript.

 We hope that F1000 Research and the Reviewer find the changes acceptable, and we look forward to publication in due course.

 None of the data has been published elsewhere. There are no conflicts of interest.

**Reviewer 1**

 The research article achieved the aim to provide a good insomnia measurement to the adolescent population. The text is objective and the procedures are adequate to questionnaire validity and reliability. Therefore, I would like to point two questions in order to improve the quality of the manuscript.

*Response: We thank the reviewer for the valuable feedback provided. We have* *addressed the comments and changed the manuscript, which we feel is now improved. Individual responses for each specific comment are outlined below*.

Major point:

 Only exploratory factor analysis was performed. The factor loadings are important to comprehend the contribution of each item into the three-factor model extracted by EFA, however, the CFA is also important to measure the factor latent structure model of the AIQ. I suggest that CFA should be included in the analysis as well as the graphic demonstration of the CFA. Lastly, it would be recommended to report the KMO and Bartlett tests to show the suitability of the sample for factor analysis in EFA and the model adjustment indexes in CFA (RMSEA, CFI, GFI, and TLI). These values ensure robustness to the models.

*Response: We thank the reviewer for the comment and have now performed the* *Kaiser-Meyer-Olkin (KMO) Measure of Sampling Adequacy test and the Bartlett test of sphericity.*

*Results show a value of 0.877 for the KMO test and a p-value <0.001 for the Bartlett test of sphericity, indicating that the sample is adequate for the Exploratory Factor Analysis.*

*Regarding the Confirmatory Factor Analysis (CFA), the main goal of our study was to present a preliminary validation of the Danish version of the Adolescent Insomnia Questionnaire (AIQ) and explore how many factors the Danish version of the AIQ has. However, our aim was not to confirm that its factor structure is the same in a different language, as it can be assessed with the CFA. In addition, the AIQ is still in an early phase of instrument development. Therefore, a CFA will be conducted when further validity evidence will be obtained on the internal structure of the scale from similar populations and contexts.*

*We have now modified the text in the methods, result and discussion sections, to strengthen these points. The text now reads:*

- *Methods section, subsection “Statistical analysis”: “* *Exploratory factor analysis (EFA) using principal factor extraction and oblique rotation was conducted to identify the factor loadings. Results of EFA were compared to those from the original development sample. The Kaiser-Meyer-Olkin (KMO) Measure of Sampling Adequacy test and Bartlett’s Test of Sphericity were performed to assess the suitability of the data for factor analysis.* *”*
- *Results section, subsection “Exploratory factor analysis”: “Results from EFA showed a three-factor solution that supported the model identified in the original development sample and accounted for 65.45% of the variance among items. Values of the rotated individual items ranged from 0.60 to 0.84 (Table 6) and were similar to those identified within the original development sample. Factor cross-loadings of the AIQ items are provided in Supplementary Table 1. The value for the KMO test was 0.877 and the p-value for the Bartlett test of sphericity was <0.001, indicating that the sample is adequate for the Exploratory Factor Analysis.”*
- *Discussion section: “Second, a CFA was not performed as our aim was not to confirm that the AIQ factor structure is the same in a different language, but to explore what the factor structure would be in such language, without making assumptions, given that the languages and culture are different. In addition, the AIQ is still in an early phase of instrument development. Therefore, future plans will be to conduct a CFA once there is further validity evidence on the internal structure of the scale from similar populations and contexts.”*

Minor points:

 In table 6, the rotated factor loading of item 1 is missing. I suggest including the lack of a social jetlag measurement as a limitation of the study. It would be relevant information to better sample description.

*Response: We thank the reviewer for the comment. The rotated factor loading of item 1 (0.78) has now been provided in Table 6. In addition, we have added the lack of a social jetlag measurement as a limitation of the study. We have now modified the text discussion section, which now reads:*

*“* *Finally, adolescence is a period where changes in bedtime occur due to both biological and social factors* *^7^* *, which, together with the effect of the inborn chronotype, might result in a social jetlag and consequential reduced sleep duration* *^32^* *. Therefore, future studies should also try to measure the social jetlag that the adolescent might be experiencing.* *”*
